# Treatment of Sensitive Dentine

**Published:** 1880-10

**Authors:** 


### EDITORIAL.

### TREATMENT OF SENSITIVE DENTINE.

In the past, the condition designated sensitive, or hyper-sensive dentine, was regarded as one of the great obstacles in the way of successful treatment and filling of decayed teeth. Indeed, so great has been the difficulty attending this condition, that multitudes of teeth have been lost through faulty operations, occasioned in part at least by this affection, and in many other instances it has prevented any effort for preservation.

In the past periods of the profession up to almost the present, a prominent enquiry has been, "How can sensitive dentine be successfully treated?"

But the intensity and frequency of that enquiry are diminishing, as true professional knowledge and skill are increasing.

A great change is now going on; indeed has already taken place.

As a knowledge of the basal principles of medical science—or dental science—is increased and made more thorough, so does ability grow and become strong. Light enables one to see his way clearly, while dimness of vision—ignorance—occasions blundering, and often fatal mistakes; and desirable results are accidental. To him who is up to the present knowledge of the growth, formation and structure of dentine, of its function, of the influences and vicissitudes to which it is subject, will there be little, if any difficulty, in deciding what ought to be done in any of the phases of the affection under consideration. And if he is at all familiar with the therapeutic and manipulative resources at his command, he will be fully able to do what he has already decided ought to be done, and that too without resort to, or reliance upon catch-penny nostrums.

The materia medica is to a remarkable extent opening up its storehouse for the dentist.

In the study of this affection it must be remembered that it presents many phases.

It is sometimes very limited, confined to a mere point, or a very superficial layer of dentine, and this may possess any degree of sensitiveness from the mildest to the most intense. Such cases are wholly dependant upon local irritants; and require for their successful treatment the removal and exclusion of the cause until nature produces restoration, which will usually be accomplished in a few days.

In the teeth there is a very great diversity of susceptibility, in structural and vital endowment, and in estimating this subject in its entirety, the infinite variety of systemic and health conditions cannot be overlooked.

Sensitiveness of the dentine is sometimes quite as much, if not more dependant upon systemic vice, than any local cause; then topical treatment alone is not sufficient, but both local and general must be employed, if the best results are to be obtained.

The most simple form of this affection, as indicated above, may be freed from irritants and protected till the part is restored; or if it is superficial, and the tooth will not be impaired by cutting it away; that may be done, but in no case should the strength of the tooth be jeopardized by cutting for this purpose.

The correct principle for the treatment of sensitive or inflamed dentine, is the removal of the affected part or its restoration to a normal condition.

Neither devitalization nor temporarily obtunding, the sensitiveness is correct in theory or practice.

Let those who would study this subject fully and profitably give attention to the following particulars, each in detail:

1st. Consider what is involved in the treatment for restoration to a normal condition.

*a.* The features and peculiarities of the case.

*b.* The opportunity available for correct treatment.

*c.* The agents best adapted to the case.

2nd. Consider the practicability of removing the affected part.

*a.* Is it superficial or very circumscribed?

*b.* Will the strength of the tooth be impaired by such removal?

*c.* The influence upon the patient.

3rd. The obtunding or temporary suspension of sensitiveness.

*a.* This is only for present convenience and does not effect a restoration.

*b.* The patient may and often does suffer great inconvenience and pain after this treatment.

*c.* In many instances the sensitiveness is increased after such treatment. Careful and intelligent discrimination may do much to avoid this.

4th. Devitalization of the affected part.

*a.* The difficulty of directing and controlling this treatment.

*b.* The danger to the parts beyond the seat of the affection.

*c.* The deterioration of the part so treated.

5th. Permitting the part to remain in its sensitive condition, and giving it protection from all irritants.

These five particulars embrace all the modes of treatment employed for sensitive dentine.

In the treatment and management of sensitive dentine the general condition of the patient must receive attention, and the more fully comprehended the better. The nature and action of local causes must be understood; the nature and action of all the available remedial agents must be well known; and when the knowledge in these different directions is attained, and in connection with a good degree of common sense there will be but little difficulty in the management of sensitive dentin e.

